# Calculating the return on investment of mobile healthcare

**DOI:** 10.1186/1741-7015-7-27

**Published:** 2009-06-02

**Authors:** Nancy E Oriol, Paul J Cote, Anthony P Vavasis, Jennifer Bennet, Darien DeLorenzo, Philip Blanc, Isaac Kohane

**Affiliations:** 1 Department of Anesthesia and Critical Care, Beth Israel Deaconess Medical Center, Harvard Medical School, Boston, MA, USA; 2 Independent Health Care Consultant, Boston, MA, USA; 3 Health Outreach to Teens, Callen-Lorde Community Health Center, New York, NY, USA; 4 The Family Van, Harvard Medical School, Boston, MA, USA; 5 Mobile Health Clinics Network, San Francisco, CA, USA; 6 Robert Wood Johnson Medical School, Camden, NJ, USA; 7 Countway Library of Medicine, Harvard Medical School, Boston, MA, USA

## Abstract

**Background:**

Mobile health clinics provide an alternative portal into the healthcare system for the medically disenfranchised, that is, people who are underinsured, uninsured or who are otherwise outside of mainstream healthcare due to issues of trust, language, immigration status or simply location. Mobile health clinics as providers of last resort are an essential component of the healthcare safety net providing prevention, screening, and appropriate triage into mainstream services. Despite the face value of providing services to underserved populations, a focused analysis of the relative value of the mobile health clinic model has not been elucidated. The question that the return on investment algorithm has been designed to answer is: can the value of the services provided by mobile health programs be quantified in terms of quality adjusted life years saved and estimated emergency department expenditures avoided?

**Methods:**

Using a sample mobile health clinic and published research that quantifies health outcomes, we developed and tested an algorithm to calculate the return on investment of a typical broad-service mobile health clinic: the relative value of mobile health clinic services = annual projected emergency department costs avoided + value of potential life years saved from the services provided. Return on investment ratio = the relative value of the mobile health clinic services/annual cost to run the mobile health clinic.

**Results:**

Based on service data provided by The Family Van for 2008 we calculated the annual cost savings from preventing emergency room visits, $3,125,668 plus the relative value of providing 7 of the top 25 priority prevention services during the same period, US$17,780,000 for a total annual value of $20,339,968. Given that the annual cost to run the program was $567,700, the calculated return on investment of The Family Van was 36:1.

**Conclusion:**

By using published data that quantify the value of prevention practices and the value of preventing unnecessary use of emergency departments, an empirical method was developed to determine the value of a typical mobile health clinic. The Family Van, a mobile health clinic that has been serving the medically disenfranchised of Boston for 16 years, was evaluated accordingly and found to have return on investment of $36 for every $1 invested in the program.

## Background

Since the 1980s, disparities in health outcomes among many US sub-populations have been well documented. The primary predictors of inadequate healthcare are racial and/or ethnic minority status, female gender, rural residence and extremes of age [1]. It is well documented that even a person's access to health insurance does not obviate the pervasiveness of unequal treatment [2]. Mobile health clinics (MHCs) serve the full spectrum of at risk populations from the disenfranchised African-American with diabetes, to the homeless person, to the child living in a rural environment who has no health insurance. They are often the provider of last resort when the mainstream system has failed to provide an environment that engenders trust or when there is no healthcare service at all.

The use of costly emergency departments (EDs) for non-urgent care by the under- and uninsured is well documented [3,4]. Less well documented (there is limited published literature in the field) is the fact that MHCs are known for serving difficult to access sub-populations, in both rural and urban areas, with cost-effective preventive approaches to healthcare [5-7]. MHCs have emerged in response to the inherent needs of local communities and have provided millions of America's most vulnerable individuals with access to healthcare. These clinics address both acute and chronic medical conditions using non-judgmental and client-led approaches, which can empower their clients to overcome stigmas against traditional healthcare institutions in order receive treatment. In addition to their role as an alternative source for healthcare delivery, MHCs also serve as intermediary portals for patients to access more mainstream services (through hospitals, community health centers, etc.).

Despite the substantial contributions made by mobile healthcare programs to the US healthcare system, evaluating the efficacy of such programs is currently difficult. Traditional research methods do not fit well in the environment needed to develop successful relationships with the clients of van programs, clients who are often marginalized or homeless or undocumented and almost always untrusting. Successful methods van workers have developed to deal with their clients, for example, the shield of anonymity that is so important to patient engagement, precludes systematic tracking of important data such as ED diversion, follow-up care, and health outcomes.

The MHC industry is at a crossroads of evolving financing strategies similar to that experienced by its healthcare predecessors: hospitals and community health centers. A driving force in this development has been the emergence of the dual imperatives of enhancing access and shifting care to more cost-effective settings. Paralleling this evolution has been a shift from a grant and/or charity-based funding structure to a negotiated exchange of payment-for-services through government and private health insurance and/or subsidies. However, to facilitate this shift in payment models, and thus an appropriate and sustainable funding stream, we must be able to quantify the value of the contributions of the MHCs and to demonstrate the potential benefit of increased investment in this system of healthcare delivery.

We hypothesize that by using published data that quantify the value of prevention practices, an empirical method can be developed to determine the efficacy of mobile healthcare programs. Specifically, mobile healthcare programs annually provide a broad array of prevention services to as many as 4 million otherwise un-served individuals. Recent, ground-breaking research from the National Commission on Prevention Priorities (NCPP) assigned a relative value to various prevention practices in terms of quality adjusted life years saved (QALYS). When combined with research that estimates the value of a statistical life year saved, and applied to mobile healthcare data, the result projects a return on investment (ROI) ratio of at least 30:1, a value both significant and compelling. We believe that this methodology can be enhanced, and applied to mobile healthcare programs across the USA to quantify the value of their current services as well as support the process of prioritization of target populations and interventions that promise the greatest return for healthcare dollars invested. We believe that such a ROI calculator will be an innovative and effective method of quantifying the value of mobile healthcare programs within the US healthcare system.

In order to test this hypothesis, we developed a 'prototype' or model. Using a sample mobile healthcare program, and published research to quantify health outcomes, we developed an algorithm to calculate the ROI of a typical broad service MHC.

## Methods

To develop the ROI prototype, the following elements formed the calculator algorithm:

1. Using sample cost and service data from a selected MHC we developed a cost baseline for a sample service population.

2. Using published data we projected QALYS for selected interventions provided within the sample MHC [8].

3. By identifying interventions within the service sample with high correlation to ED visits, we estimated the number of ED visits prevented by mobile healthcare interventions.

4. Using published data we applied a value of statistical life years saved (VSLYS) and avoidable cost of ED visits prevented to quantify the ROI of our sample mobile healthcare program.

Sample cost and service data for the Harvard Medical School-sponsored 'The Family Van' for the academic year 2008 were used to provide clinic specific cost data to generate a per visit cost to compare with ED per visit costs to calculate 'cost avoided' as a measure of cost effectiveness. Data from the 2007 Massachusetts Division of Health Care Finance and Policy publication *Analysis of 2005 Preventable Emergency Department Visits *was used as the source for the cost of preventable ED visits [9], Actual service data from The Family Van were extracted to match the intervention categories used by the NCPP to allow the estimation of QALYS and the calculation of their projected 'value' [8].

It should be noted that in addition to medical screenings, education and counseling, individuals served by The Family Van access a broad array of services directly through the van, and/or its formal linkages with Neighborhood Health Centers (the effectiveness of this linkage is reinforced by The Family Van's anecdotally high referral follow-up rate).

The NCPP research provided the pivotal estimates of outcome utilities in terms of QALYS. However, to be adapted for use in calculating a financial return on investment, the QALYS metric needed to be converted into financial terms. In order to address this issue, we used the 1990 research of Tolley *et al. *[10] to provide a dollar value of the QALYS saved, using Tolley's $70,000 VSLYS.

## Results

As a result of applying the methods outlined, we calculated the ROI of the sample mobile healthcare program to be 36:1.

### Annual projected ED visit costs avoided (Table 1)

**Table 1:** Cost savings from preventing unnecessary emergency department visits.

	Annual expenses ($)
Annual cost of mobile health clinic	565,700

Total annual visits	4,848

Total visits discounted by 20%	3,878

Cost per visit	117

Cost of preventable emergency department visit [9]	923

Cost avoided per mobile clinic visit	806

Annual projected costs avoided	3,125,668

The Family Van expenses versus costs for non-emergent and emergent but primary care treatable emergency department visits.

In academic year 2008 The Family Van expenses were $565,700. There were 4,848 visits to the Van. These visits are discounted by 20% to allow for interventions within the service sample not highly correlated to ED visits. The resulting 3,878 visits would presumably have resulted in a preventable ED visit had the Van not been available [9], the remaining 3,878 visits cost $117 each. The cost of an ED visit for non-emergent and emergent but primary care treatable ED visit was $923. The annual projected costs avoided by providing services on the Van rather than in the ED were estimated to be $3,125,668. It was assumed that most patients receiving care from the Van would have otherwise gone to the ED based on the fact that less than 50% of Van patients reported having a personal care physician, combined with their effective decision to seek an alternative to other outpatient options as evidenced by their choice of MHC services in the first place.

### Total annual value of life years saved (Table 2)

**Table 2:** Potential value of selected prevention services provided by The Family Van.

**Recommended interventions **[8]	**Actual number of services provided**	**Total number of QALYS saved**	Estimated value of QALYS saved* ($)
Hypertension screening and treatment	1,693	152	10,640,000

Vision screening	123	3	210,000

Cholesterol screening and treatment	616	55	385,000

Obesity screening	949	27	1,890.000

Depression screening	110	10	700,000

Diabetes screening	1039	4	280,000

Diet counseling	796	3	210,000

Total annual value of life years saved	5,326	254	17,780,000

Visits are distributed by risk for initial visits only (42% of total). More than one service may have been provided at each visit. *Value of statistical life year, $70,000 [10].

QALYS = quality adjusted life years saved.

In addition to preventing unnecessary ED visits, this mobile clinic also provided several of the NCPP recommended prevention services. The NCPP recommended prevention services provided include: hypertension screening, vision screening, cholesterol screening, obesity screening, depression screening, diabetes screening and diet counseling. Only first-time visits were counted and the NCPP estimations assume a 30% non-compliance with treatment [8]. Cost-benefit and effectiveness values from NCPP ranking of clinical preventive services project that this mobile clinic is responsible for 254 QALYS saved. Applying a dollar value to a life year of $70,000 yields a cost savings of $20,339.968 [10].

Individuals in need of follow-up were served either directly by the MHC, or through its formal linkages with Neighborhood Health Centers.

### Annual ROI of The Family Van program (Table 3)

**Table 3:** Prototype return on investment 'Report Card'.

Annual funds invested for The Family Van services	$565,700
Cost avoided by preventing emergency department visits	$3,125,668

Value of potential life years saved by The Family Van services	$17,780,000

Annual return on investment of The Family Van	$20,339,968

Return on investment ratio for The Family Van	36

Annual funds invested for The Family Van services totaled $565,700. ED costs avoided totaled $3,125,668. The value of potential life years saved by the Van services totaled $17,780,000. With these inputs, the resulting annual ROI of The Family Van is US$20,339,968 for a ROI ratio of 36:1.

## Discussion

By using a sample mobile healthcare program and published research we were able to calculate a ROI value that indicates that for every dollar invested in funding for that mobile healthcare program $36 were returned in combined value of life years saved, and ED costs avoided. In that we have used an ROI-based metric to determine the effectiveness of preventive services, while proposing to decrease both the incidence and economic burden of preventable diseases as a result of rendering such services, our results echo the findings of a recent study conducted by Trust for America's Health in partnership with the New York Academy of Medicine, the Robert Wood Johnson Foundation, and The California Endowment and Prevention. This study calculated the ROI to state healthcare systems of small local prevention programs. Reassuringly, data from this collaborative effort detailed healthcare savings of up to $16 billion annually with only marginal investments in preventive services per person per year [11].

There are several limitations to our study. It should be stated at the outset that adapting the 'population-based' research of the NCPP series to derive individual estimates of QALYS is specifically discouraged in the NCPP research. However, as acknowledged in the NCPP research, the disproportionate vulnerability of the mobile healthcare target population makes the adaptation of these population values reasonable for the 'prototype' purposes. In addition, we used the lower estimate of life years saved in all cases [8]. Nonetheless two assumptions require validation in the near future.

1. The assumption that MHC preventive services interventions are no less effective in preventing more expensive uses of healthcare than those reported in other studies [2].

2. The distribution of healthcare status among the MHC population provides at least equivalent opportunities for effective prevention.

Further limitations include the fact that the ROI calculation applies cost data from 2005 to 2008 service data, as well as using research values for QALYS from a 2004, and for VSLYS dollar values from 1990. Although the result of this calculation, assuming annual growth in healthcare costs, arguably results in a more conservative estimate of the return on investment, the data used would optimally be from the same year. This limitation should be addressed in future iterations of this project.

In addition, it should be noted that it is assumed that the ED would be the only alternative source of care for MHC clients. This assumption needs to be based on more verifiable data in future iterations of this project.

The conversion of quality outcome data to a dollar value is hardly unique to this effort and has a long pedigree. Many public health projects must estimate the cost of inaction in terms of life years lost, and it was the review of research supporting the national Clean Air Act that yielded the most applicable research on determining the value of a statistical life year. Due to its use of quality adjusted life years, and because it calculates the lowest value for a statistical life year, we selected the Tolley *et al. *model [10].

A priority of future efforts will be to revisit the estimates that we have borrowed from other studies for QALYS and VSLYS related to MHC interventions and provide more robust lower bounds for the populations specifically served by the MHCs.

The generalization of this approach will require the adaptation of the calculator algorithm to several different MHC programs. This in turn will require the development and adoption of a uniform data collection tool, service taxonomy, and reporting channel to provide the evidence with which to drive further resource allocation and decision making for the underserved communities reached by MHCs.

## Conclusion

By using a sample mobile healthcare program and published research we were able to calculate a ROI value that suggests that, for every dollar invested in funding for that mobile healthcare program, $36 may be returned in combined ED costs avoided, and the value of life years saved.

The implications of this ROI should be used to promote the effectiveness of the program model among healthcare policy-makers, as well as to seek funding to create a continuous self-assessment tool for use by mobile healthcare providers to promote healthcare practices that provide the greatest healthcare benefit for every healthcare dollar invested.

Ultimately the calculator, when paired with services data rendered by each of the MHCs across the country, should serve as a powerful communication tool, whereby individual providers will be able to enter their own data, calculate ROI, compare their results to industry counterparts, establish benchmarks, and learn and change accordingly.

## Abbreviations

ED: emergency department; MHC: mobile health clinics; NCPP: National Commission on Prevention Priorities; QALYS: quality adjusted life years saved; ROI: return on investment; VSLYS: value of statistical life years saved

## Competing interests

All the authors except PB and IK work with MHCs. Proving the value of mobile healthcare will be of benefit as it will prove the usefulness of this form of service delivery. All the authors have applied for funding to further develop this project and build a web-based calculator that will be accessible to all mobile programs.

## Authors' contributions

NEO initially conceived the idea of a ROI calculator for mobile healthcare and prevention services. She is founder of The Family Van, and was first author in drafting the manuscript and had final approval. PJC, the former Massachusetts Commissioner of Public Health, designed the calculator algorithm, performed the statistical analysis and background investigation to develop the calculator, made significant contributions to the manuscript and had final approval. APV was the co-creator of the idea of calculating a ROI for mobile healthcare and prevention services, helped to draft the manuscript and had final approval for the manuscript. JB, Executive Director of The Family Van, collated and provided the cost and service data to test the algorithm and helped to draft the manuscript. DD provided expertise related to the national mobile healthcare service sector and helped to draft the manuscript. PB provided significant help in reviewing the literature and drafting the manuscript. IK provided expertise on health informatics and the use of, and assumptions about QALYS and VSLY, helped to draft the manuscript and had final approval.

## Pre-publication history

The pre-publication history for this paper can be accessed here:


